# Clamping-mediated incorporation of single-stranded DNA with concomitant DNA synthesis by Taq polymerase involves nick-translation

**DOI:** 10.1038/s41598-024-52095-3

**Published:** 2024-01-23

**Authors:** Yoshiyuki Ohtsubo, Syoutaro Kawahara, Yuji Nagata

**Affiliations:** Department of Molecular and Chemical Life Sciences, Graduate School of Life Sciences, Tohoku University, 2-1-1 Katahira, Sendai, 980-8577 Japan

**Keywords:** Biochemistry, DNA, Enzymes

## Abstract

The development and characterization of a new enzyme reaction contribute to advancements in modern biotechnology. Here, we report a novel CIS (clamping-mediated incorporation of single-stranded DNA with concomitant DNA synthesis) reaction catalyzed by Taq polymerase. In the reaction, a single-stranded DNA (ssDNA) with 3′ Cs is attached with a preformed 3′ G-tail of double-stranded DNA (dsDNA); DNA syntheses starting from both 3′ ends result in the incorporation of ssDNA. A 3′ G-tail length of 3 nucleotides adequately supports this reaction, indicating that Taq polymerase can clump short Watson–Crick base pairs as short as three pairs and use them to initiate DNA polymerization. The reverse transcriptase from Molony murine leukemia virus catalyzes strand displacement synthesis and produces flapped-end DNA, whereas the reaction by Taq polymerase involves the nick translation. These new reaction properties may be beneficial for the development of new molecular tools applicable in various fields. Apart from its CIS reaction activity, we also report that Taq polymerase has the undesirable characteristic of removing 5' fluorescent labels from dsDNA. This characteristic may have compromised various experiments involving the preparation of fluorescently-labeled dsDNA by PCR for a long time.

## Introduction

Several enzymatic reactions are often used in various biological experiments. Enzymes that recognize and catalyze DNA substrates are essential molecular tools frequently used in modern biotechnology.

Several DNA polymerases are used in the tailing reaction that adds one or more nucleotides to the 3′ end of double-stranded DNA or DNA-RNA hybrids, forming a protruding end. The well-known Taq polymerase developed from *Thermus aquaticus*^1^ mediates this reaction and creates a 3′ protruding end consisting of a single adenosine monophosphate. In contrast, terminal deoxynucleotidyltransferase adds a large number of nucleotides in an uncontrolled manner^2^. We previously reported that Molony murine leukemia virus reverse transcriptase (MMLV-RT)^3^ is substantially efficient in tailing double-stranded DNA (dsDNA). MMLV-RT can add several As, Cs, Gs, or Ts to the 3′ end of DNA molecules^4^. Moreover, specific tailing enhancers for C, G, and T-tailing reactions, such as deoxyguanosine, deoxycytidine, and adenosine, respectively, which facilitate the addition of 3–5 nucleotides (As, Cs, Gs, or Ts), were also reported (see reference^5^ for proposed mechanisms of how the enhancers work). The protruding tails are utilized for various purposes, including TA cloning^6^, CG cloning^7^, DNA labeling^8,9^, and the CIS reaction.

Besides the strong tailing capacity, the unique effectiveness of MMLV-RT includes its catalytic activity in the template-switching reaction. It appends a few Cs to the 3′ end of the newly synthesized DNA at the end of the RNA template. In the presence of G-tailed single-strand DNA (ssDNA), MMLV-RT can switch the template and polymerize the complementary strand of incoming ssDNA^10,11^. This template-switching reaction has been widely used in various studies to analyze RNA-seq^12^.

Some reverse transcriptases, including MMLV-RT, can incorporate single-stranded DNA (ssDNA) into double-stranded DNA (dsDNA) based on complementarity as short as one nucleotide. These reverse transcriptases are considered to have clumping activity, an ability to stabilize the very short complementarity and aid the synthesis of the complementary strand of ssDNA^13–15^. However, the reaction was rarely used owing to its limited efficiency until we developed a method to create long protruding 3′ ends^5^. The long tail enabled the formation of thermodynamically stable Watson–Crick base pairings and improved the reaction efficiency up to approximately 100%. We marked this reaction as the CIS (clamping-mediated incorporation of single-stranded DNA with concomitant DNA synthesis) reaction^16^.

**Figure 1:**
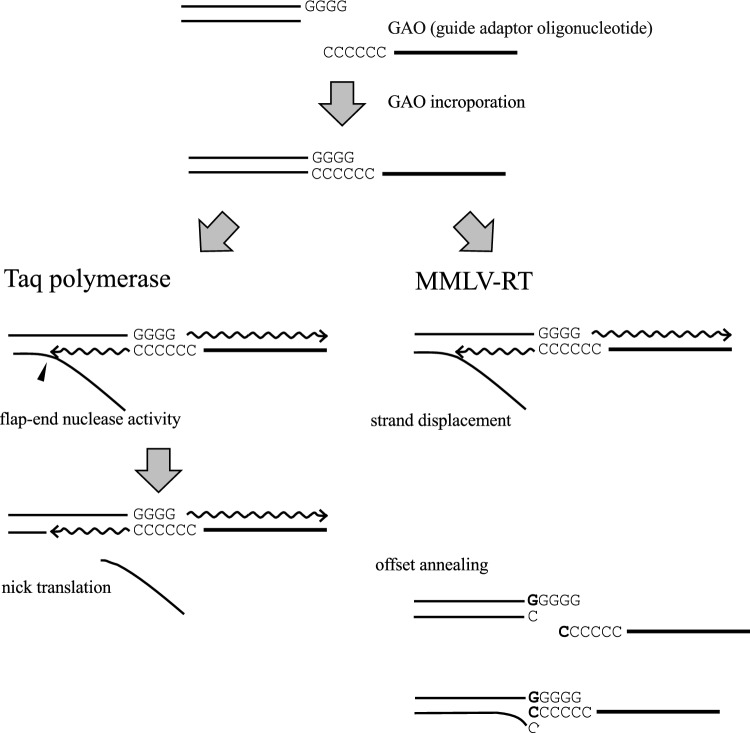
Schematic representation of CIS reactions mediated by Taq polymerase and MMLV-RT. To a dsDNA substrate carrying a 3′ G-tail, ssDNA with 3′ Cs (GAO; guide adaptor oligonucleotide) was attached via Watson–Crick base pairings. Taq polymerase and MMLV-RT catalyze DNA polymerization starting from the 3′ end of the G-tail, as well as the 3′ end of GAO, resulting in the incorporation of GAO. While Taq polymerase catalyzes nick translation (left), MMLV-RT causes strand displacement synthesis (right). In this study, MMLV-RT was postulated to have undergone offset annealing and extension (bottom right).

The MMLV-RT-mediated CIS reaction using dsDNA with four Gs at the tail and ssDNA with multiple Cs at the 3′ end was highly effective, and all substrate DNAs were converted into products^16,17^. Notably, the ssDNA can carry a stretch of Ns, which can be used as a unique molecular identification tag^18–20^. These tailing and CIS reactions are highly efficient and can incorporate any desired sequence (up to 70 nucleotides long) into the ends of dsDNA with nearly 100% efficiency^16^. A potential disadvantage of the use of MMLV-RT in CIS reaction is that it catalyzes strand-displacement synthesis producing flapped-end DNA (Fig. 1)^16^.

The addition of four Gs to the 3′ end of double-stranded DNA represents a novel reaction^5^. Methods for utilizing such ends, for example, in the labeling of DNA ends with UMIs, have not been fully explored. To pioneer new applications and to investigate whether clumping activity is unique to some reverse transcriptases, we evaluated the efficacies of different DNA polymerases in catalyzing CIS reactions. In this study, we investigated the ability of Taq polymerase to catalyze a different type of CIS reaction that develops nick-translated products^21^ and described the optimum conditions required for such reactions.

## Materials and methods

### Reagents

Taq polymerase with the reaction buffer was obtained from New England Biolabs (Ipswich, Massachusetts). DNA ligation mix, Exonuclease I, and T4 DNA polymerase were purchased from TAKARA (Shiga, Japan).

### DNA length analysis using a capillary sequencer

Length of DNA was analyzed as described previously^4^. To prepare HiDi-LIZ1200, 1 ml of HiDi formamide was mixed with 10 µL of GeneScan-1200 LIZ Size Standard (Thermo Fisher Scientific, USA) containing 68 fragments of known sizes. Subsequently, 0.2–1.0 µl of the reaction mixture or diluted purified DNA samples not exceeding 0.5 fmol were added to 12.5 µl of HiDi-LIZ1200, as appropriate. The samples were analyzed using a 3130xl Genetic Analyzer (Thermo Fisher Scientific, Massachusetts, USA) with a 50-cm capillary array and POP7 polymer. The data obtained were analyzed using the TraceViewerForMolecularBiology software (our product available at App Store, Apple Inc., California)^4^; two LIZ bands were chosen to calibrate the electropherogram. Peak areas were determined using the software.

### Preparation of DNA substrates

DNA substrate 1 (33G1, 33G2, 33G3, and 33G4) was adopted from a previous report^16^. To prepare substrate 2, SA680 (FAM-TCGTCGGCAGCGTCAGATGTGTATAAGAGACAGGGGG) and SA760 (5′-GAGTTCAGACGTGTGCTCTTCCGATCTCCCCCCCTGTCTCTTATACACATCTGACGCTGCCGACGA-3′) were annealed. Substrate 3 was prepared by annealing SA761 (5′-CTGTCTCTTATACACATCTGACGCTGCCGACGA-3′-FAM) and SA763 (5′-TCGTCGGCAGCGTCAGATGTGTATAAGAGACAGGGGG-3′) and purified using polyacrylamide gel electrophoresis.

### Taq and MMLV-RT CIS reactions

A total volume of 10 µL CIS reaction mixture contained 10 fmol DNA substrate, 0.25 mM each of dNTPs (TAKARA, Kyoto, Japan), and 2 pmol guide adaptor oligonucleotide (GAO). For the Taq CIS reaction, we used 0.5–1.25 units of Taq polymerase (0.1–0.25 µl). For the MMLV-RT CIS reaction, 50 units of MMLV-RT (0.25 µl) (Nippon Gene, Tokyo, Japan) were used with the buffer containing 50 mM Tris–HCl pH 8.3, 75 mM, KCl, 6 mM MgCl_2_, and 2 mM DTT. To initiate the reactions, after incubation at 37 °C or temperatures as indicated, components of the reaction mixture were added to the premixed GAO and substrate DNA.

### Other enzyme reactions

For the Exonuclease I reaction, 0.1 µl of the enzyme solution was added to the post-CIS reaction mixture and incubated for 3–5 min at 37 °C. For the blunting reaction using T4 DNA polymerase, the DNA in the post-CIS reaction mixture was column-purified and then used for the reaction.

### DNA column purification

For DNA fragment purification, we used PCR Purification Kit (HiYield™ Gel/PCR DNA Fragments Extraction Kit) from RBC Bioscience.

## Results

### Taq DNA polymerase catalyzes the CIS reaction

We evaluated the efficiencies of several polymerases, including Taq polymerase, KOD DNA polymerase^22^, DNA polymerase I, Bst DNA polymerase, and a mutant Klenow fragment that lacks 3′ → 5′ exonuclease activity in the CIS reaction. Notably, only Taq polymerase and Klenow fragment (3′ → 5′ exo-) were sufficiently effective in conducting the CIS reaction. Taq polymerase conducts a unique CIS reaction as described in the text; hence, we focused on this enzyme. Figure 1 (top to left) displays the overall features of the CIS reaction mediated by Taq polymerase. In this reaction, a single-stranded DNA called a guide adaptor oligonucleotide (GAO) anneals to the 3′ protruding G-tail of a double-stranded DNA. Subsequently, Taq polymerase catalyzes the synthesis of the complementary strand, starting from the 3′ OH end of the protruding G and the 3′ end of GAO. As the GAO strand extends, the strand ahead of the polymerase is degraded by the flap-end nuclease activity of Taq polymerase^21^, leading to the nick translation^23^. In contrast, displacement synthesis occurs in the previously known reaction mediated by MMLV-RT, which lacks the 5′ → 3′ exonuclease activity (top to right)^16^.

The following data demonstrate these features and optimal conditions for the reaction with Taq polymerase.

### Top strand extension along with the GAO by Taq polymerase

To prepare the CIS reaction substrate (substrate 1), we annealed a 5′-FAM-labeled oligonucleotide SA680 (37 nucleotides) with SA659 (33 nucleotides). Substrate 1 was a 33 bp double-stranded DNA with four 3′-extruding Gs, which is identical to the previously reported 33G4^16^. We annealed SA680 with SA760 (66 nucleotides) to prepare substrate 2 for the Taq extension reaction.

**Figure 2:**
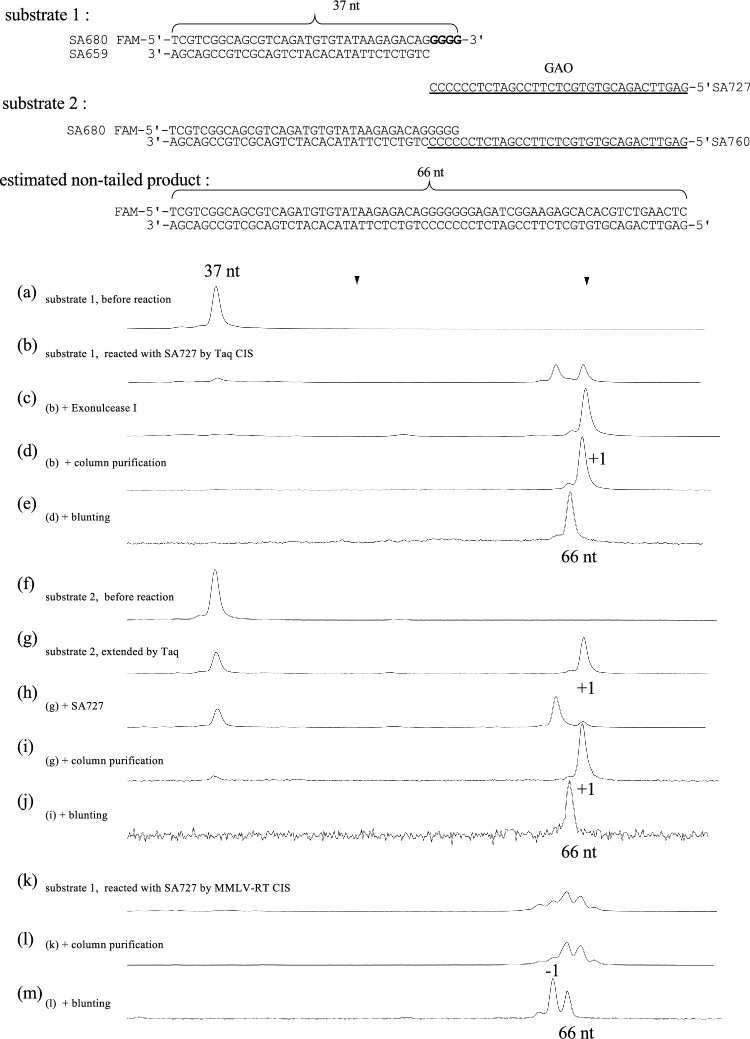
Taq polymerase conducts the CIS reaction. To a mixture of substrate 1 (top) and GAO (SA727), dNTP and Taq polymerase were added to initiate the CIS reaction. A fraction of the reaction mixture was sampled for the HiDi LIZ1200 before (a) and after the CIS reaction (b). DNAs in the post-reaction mixture were treated with Exonuclease I (c) or purified by column purification (d). The purified sample was used for the DNA blunting reaction with T4 DNA polymerase (e). Substrate 2 was used as a control for the canonical Taq extension reaction. Samples were collected before (f) and after (g) the Taq extension. The addition of SA727 to the post-reaction mixture (h) gave an electropherogram similar to that in (b). The DNAs in the post-reaction mixture were purified by column purification (i) and subjected to blunting reaction (j). Panels k, l, and m show the MMLV-RT CIS reaction products immediately after reaction (k), post-reaction after column purification (l), and after the blunting reaction (m). Two triangles indicate the LIZ size marker peaks (not shown) used to calibrate the electropherograms. An estimated non-tailed product of 66 nucleotides is indicated.

GAO (SA727 with six Cs added at its 3′ end), Taq polymerase, and dNTPs were added to substrate 1 and incubated. The change in FAM-labeled strand length was analyzed using a capillary sequencer under denaturing conditions. As compared to the initial substrate (Fig. 2a), the Taq treatment extended the FAM-labeled strand (Fig. 2b). However, the peak pattern observed when the reaction solution was directly sampled was unexpected and showed two separated peaks (Fig. 2b). This unexpected pattern was likely due to the interference of the residual GAO with the FAM-labeled product DNA during capillary electrophoresis. To support this, degradation of the residual GAO by Exonuclease I (Fig. 2c) or removal by column purification (Fig. 2d) resulted in a single major peak. This interference was also verified by the reaction using substrate 2 (Fig. 2f) and Taq polymerase (Fig. 2g) and the subsequent addition of SA727 (Fig. 2h). The sizes of the two reaction products were indistinguishable (Fig. 2d and i), and the subsequent blunting reaction shifted the peak to the left by one nucleotide, indicating their identical sizes (Fig. 2e and j). The shift reflects the removal of a single A overhang appended by Taq polymerase.

As a reference, the MMLV-RT CIS reaction products before (Fig. 2k) and after (Fig. 2l) the column purification are shown. Notably, the blunting reaction with T4 DNA polymerase presented a major peak representing a strand size one nucleotide shorter than expected (labeled as -1), which is discussed later in the text.

### GAO strand elongation and nick translation

**Figure 3:**
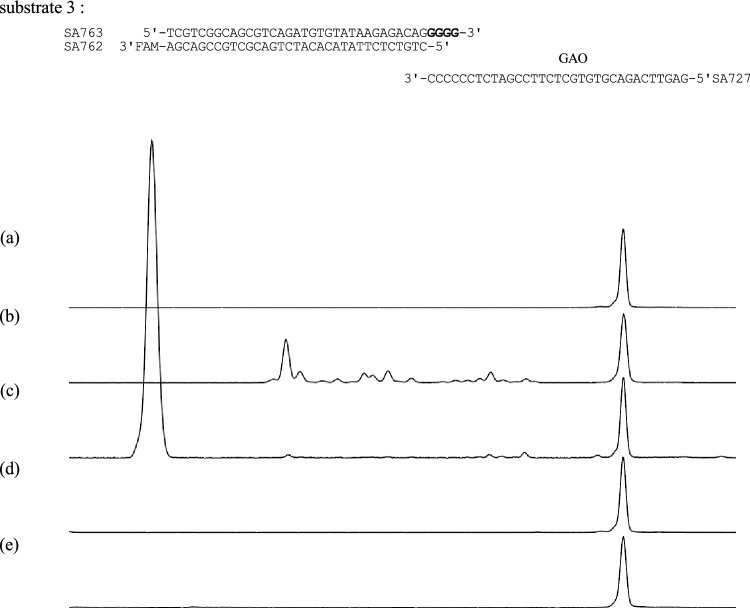
Taq polymerase-mediated CIS reaction causes nick translation. Substrate 3 carrying FAM at the 3′ end of the bottom strand (top) was analyzed before (a) and after the Taq CIS reaction, followed by phenol/chloroform/isoamyl alcohol treatment and ethanol precipitation (b). The sample shown in (b) was treated with exonuclease I to digest the single-stranded DNA (c). Short fragments were not observed when substrate 3 was incubated with Taq polymerase in the absence of GAO (d) or when MMLV-RT was used for the CIS reaction (e).

Taq polymerase has an intrinsic structure-dependent endonuclease activity that cleaves the displaced strand (also referred to as flapped-end DNA), exhibiting a phenomenon known as nick translation. We examined whether nick translation occurs during GAO strand elongation. We prepared a substrate DNA (substrate 3), which was FAM-labeled at the 3′ end of the bottom strand (Fig. 3a), and conducted a CIS reaction using Taq polymerase. Consequently, a group of short fragments was produced (Fig. 3b), indicating that the FAM-labeled strand was shortened by nick translation. The short fragments were sensitive to Exonuclease I (Fig. 3c), indicating that they are single-stranded DNAs that dissociate from double-stranded DNA before cleavage by the flapped-end nuclease activity of the Taq polymerase. Such short fragments were not generated if the substrate DNA was incubated in the absence of GAO (Fig. 3d) or MMLV-RT was used (Fig. 3e). Based on these results and previously known characteristics of Taq polymerase, we conclude that nick translation occurs at the bottom strand in the Taq polymerase-mediated CIS reaction.

### Effect of reaction temperature

**Figure 4:**
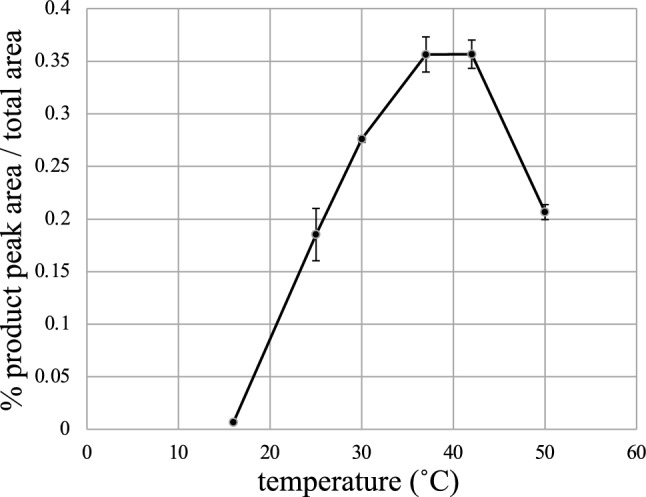
Optimal temperature for the Taq CIS reaction. Substrate 1 (10 fmol) was reacted with 2 pmol GAO (SA727) in a 10 µL reaction mixture using Taq polymerase (0.5 unit). The reactions were conducted at different temperatures for 10 min and analyzed by capillary electrophoresis using a 3130xl capillary sequencer. The ratio of the CIS reaction product peak area to the total peak area was calculated. The values represent the average of three independent reactions, and the error bars represent standard deviations.

To establish the CIS reaction with Taq polymerase and enhance its usefulness as a DNA-manipulating tool, we optimized the reaction temperature. The CIS reaction product was effectively generated at a temperature ranging between 37 and 42 °C (Fig. 4). At higher temperatures (42 °C and 50 °C), a broad peak possibly representing FAM plus one nucleotide was observed (see Fig. 6a,b and relevant discussion).

### 3' G-tailing length dependency

**Figure 5:**
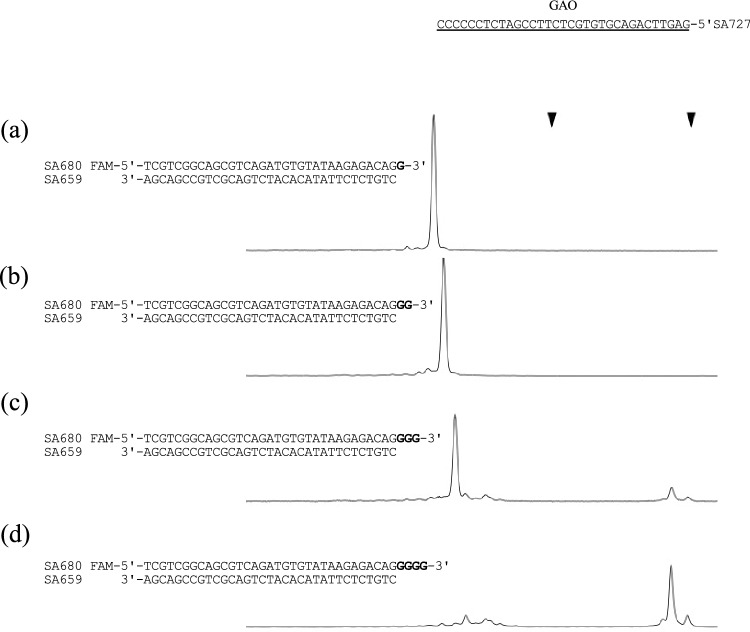
3′-G tail length of three is the minimum requirement for the CIS Taq reaction. Four different substrates (a–d) with one to four extruding Gs were used for the CIS reaction with SA727 (GAO). After 10 min at 30 °C, the reaction mixture was analyzed by capillary electrophoresis. Two triangles indicate the LIZ size marker peaks (not shown) used to calibrate the electropherograms.

We analyzed whether the length of the 3′ G-tail influences the efficiency of the reaction (Fig. 5). We used four substrate DNAs with G-tails ranging from one to four nucleotides in length and conducted the Taq CIS reaction at 30 °C. G-tails of 3 and 4 nucleotides facilitated the generation of a product, and four-nucleotide-long G-tailed substrate exhibited distinctly higher efficiency. In contrast, no CIS reaction products were obtained when the G-tails of nucleotides 1 and 2 were used.

## Discussion

In this study, we demonstrated that well-known and widely used Taq DNA polymerase catalyzes the CIS reaction. This reaction is similar to but different from that catalyzed by MMLV-RT, as Taq polymerase and MMLV-RT produce nicked (Fig. 1) and flapped-end products, respectively.

### Usage of CIS reaction in DNA labeling with unique molecular identifiers (UMIs)

Our development of the CIS reaction will benefit modern biotechnologies by providing a means to label DNA ends with unique molecular identifiers (UMIs). The CIS reaction can be used for the efficient labeling of DNA ends with desired DNA sequences carrying UMIs.

The UMI-labeling of DNA ends is key technology to the development of a method called qTnSeq for quantitative analysis of Tn mutant libraries. In qTnSeq, DNA obtained from a Tn mutant library was fragmented by sonication. The generated DNA ends were end-repaired and G-tailed using MMLV-RT in the presence of deoxycytidine, a tailing enhancer. Subsequently, for Illumina analysis, a CIS reaction was conducted to append the adapter sequence to the tailed ends; and then the NGS sequence library is prepared by PCR.

If a portion of the GAO contains a random sequence (e.g., N13), upon acquisition of NGS reads, sequences with the same UMI can be identified as originating from the same DNA molecule, thereby preventing overcounting of duplicate reads. Using UMI, Tn mutant libraries can be analyzed with high quantitative accuracy. The usefulness of UMI labeling of DNA ends in qTnSeq will be published elsewhere.

### General guidance for preparation of CIS reaction substrate

A proper 3′ G-tailing of the substrate DNA is crucial for the CIS reaction. The G-tailing reaction conducted in the presence of the specific enhancer deoxycytidine typically appends 4–5 Gs. However, the tailing reaction requires a 3′ OH group in the substrate DNA. The DNA 3′ ends generated by sonication have long been misunderstood as primarily carrying a hydroxyl group. We previously reported that only approximately 20% of sonication-generated DNA 3′ ends carry hydroxyl groups, whereas approximately 50% and 30% of them carry 3′-phosphate groups or unknown structures, respectively^17^. The possible unknown structures may include a 2',3'-double bond, which could be formed upon breakage at an abasic site, although this remains to be fully elucidated^17^. To convert DNA termini into hydroxyl groups, we routinely use SB-repairing (scrap-and-build repair), in which 3′ → 5′ activity of exonuclease III is counter balanced by the DNA synthesis activity of T4 DNA polymerase^17^.

### Advantages of CIS reaction conducted by Taq polymerase

We have identified several advantages of the use of Taq polymerase in CIS reactions compared to the use of MMLV-RT. First, when MMLV-RT is used, dsDNA with a displaced strand is obtained as the CIS reaction product. It would be difficult to estimate such DNA size by electrophoresis. In contrast, in Taq polymerase-based CIS reaction, the size can be reliably estimated by electrophoresis. Second, MMLV-RT appends GAO recursively and produces a concatenated end. Owing to the tailing activity of MMLV-RT, it appends some nucleotides to the 3′ end after synthesizing the complementary strand of GAO, and another GAO can be incorporated, resulting in a GAO-concatenated end. Although placing a biotin moiety at the 5′ end of GAO prevents concatemer formation by MMLV-RT^16^, the use of Taq polymerase, which mostly appends a single A without concatemer formation, is favorable. Third, as shown in Fig. 2m, the CIS reaction by MMLV-RT yielded a shorter product than the expected size. In the CIS reaction, we expected that the last 3′ cytosine base of GAO would anneal to the first guanine base of the tail. The unexpected short (-1) product peak of the MMLV-RT-mediated CIS reaction is possibly attributable to offset annealing of GAO, in which the last 5′ cytosine base of the dsDNA is displaced by the 3′ cytosine base of GAO (Fig. 1 right bottom). The Taq-polymerase-mediated CIS reaction product has less possibility to be affected by offset annealing, which could compromise downstream in silico analysis. Lastly, Taq polymerase is considered to be significantly more efficient DNA polymerase compared to MMLV-RT. Those are several advantages of using Taq polymerase in CIS reactions compared to the use of MMLV-RT. On the other hand, the use of MMLV-RT in CIS reactions also has its own unique advantages, such as its faster reaction speed and its strong catalytic activity towards short G-tails of 1 or 2 Gs. The insights provided in this discussion will guide researchers in selecting either one of the two polymerases for their CIS reactions.

Those advantages discussed above highlights the novelty of the CIS reaction catalyzed by Taq polymerase, which would be useful in future research activities and potential practical applications.

### GAO Interfering with the FAM-labeled DNA in capillary electrophoresis

When the post-CIS reaction solution was directly sampled and analyzed using a capillary sequencer in HiDi formamide, one unknown peak was observed in addition to the expected product peak (Fig. 2b). We later found that the commercially available size standard obtained from Promega (CLS600), which is designed to be used under denaturing conditions, contains counteracting oligonucleotides that suppress the extra peaks^24^. In this study, we evaluated and confirmed the interference of GAO affecting the outcome of the capillary electrophoresis of FAM-labeled strands. DNA analysis under denaturing conditions using capillary sequencers is convenient and effectively used in various analyses^4,5,16,17,25–27^. This information regarding the interference of single-stranded DNA in electrophoresis would be crucial for its further applications in various analyses.

### Nature of the broad peak observed at higher temperatures

**Figure 6:**
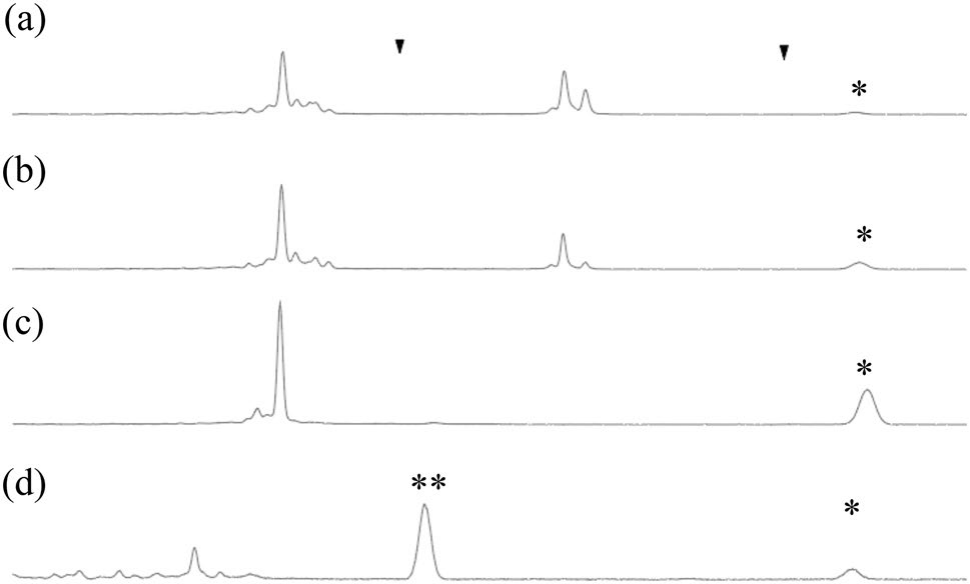
Taq polymerase cleaves off 5′ FAM independent of the CIS reaction. Taq CIS reaction of substrate 1 with GAO (SA727) produced an unknown broad peak (marked with “*”) at elevated temperatures of 42 °C (a) and 50 °C (b). A peak was observed when substrate 1 was incubated with Taq polymerase in the absence of SA727 (c). Substrate 1 treated with T4 DNA polymerase in the absence of dNTP also produced the same peak (indicated by “*”) and an additional peak (indicated by “**”) (d). The peaks indicated by “*” and “**” represent FAM-5′-T-3′ and FAM-5′-TC-3′, respectively. Two triangles indicate the LIZ size marker peaks (not shown) used to calibrate the electropherograms.

A broad peak was observed when the Taq polymerase-mediated CIS reaction was conducted at 42 °C or 50 °C (Fig. 6a,b indicated by *). As it was also observed when 5′-FAM-labeled dsDNA was incubated with Taq polymerase in the absence of GAO at 50 °C (Fig. 6c), it must not be related to the CIS reaction. Furthermore, a peak at the same location was observed when substrate 1 was treated with T4 DNA polymerase in the absence of dNTPs (T4 DNA polymerase degraded dsDNA into mononucleotides in the absence of dNTPs) (Fig. 6d).

This broad peak may represent the FAM with a T nucleotide (FAM-5′-T-3′). It is possible that at elevated temperatures, two strands of DNA locally melt at the terminus and form a structure recognized and cleaved by the Taq polymerase with its flap-end nuclease activity^21^. The resulting FAM-5′-T-3′ may migrate slowly owing to its large mass compared to its electric charge. Although further investigation is needed to understand the mechanism by which Taq polymerase produces this peak, we recommend avoiding the use of Taq polymerase when preparing double-stranded DNA with 5′ fluorescent labels for downstream applications such as gel-shift assays or FISH experiments.

### Clumping activity of Taq polymerase

The unusual activity of Taq polymerase demonstrated in this study indicates that a 3–4 nucleotide long G-tail was sufficient to accept C-tailed single-stranded DNA, and DNA polymerization was primed and initiated. Hence, Taq polymerase has a clumping activity. Since Watson–Crick base pairing of four G-C pairs is not stable at 42 °C or 50 °C, the base-stacking interaction between the base at the 3′ end of GAO and the 5′ end of the accepting dsDNA was possibly involved in this reaction.

In conclusion, we believe that our findings will aid the establishment of new molecular techniques applicable in various fields of research in the future. It is possible to design and obtain a mutant Taq polymerase that has increased clumping activity and can accept dsDNA with shorter G-tails as a substrate.

## Data Availability

The original data supporting the findings of this study are available upon reasonable request. To request the data, please contact Yoshiyuki Ohtsubo at yoshiyuki.ohtsubo.a6@tohoku.ac.jp. The data will be made available under the condition that it is used only for non-commercial research purposes and that any publications or presentations resulting from the use of the data cite this study as the source of the data.
